# (*E*)-3-Phenyl-3-(3-phenyl-1*H*-1-pyrazol­yl)-2-propenal

**DOI:** 10.1107/S1600536810004332

**Published:** 2010-02-10

**Authors:** V. Susindran, S. Athimoolam, S. Asath Bahadur, B. Sridhar, R. Manikannan, S. Muthusubramanian

**Affiliations:** a Department of Lighthouses & Lightships, Ministry of Shipping, Nagapattinam Lighthouse & DGPS station, Nagapattinam 611 001, India; b Department of Physics, University College of Engineering Nagercoil, Anna University Tirunelveli, Nagercoil 629 004, India; c Department of Physics, Kalasalingam University, Anand Nagar, Krishnan Koil 626 190, India; d Laboratory of X-ray Crystallography, Indian Institute of Chemical Technology, Hyderabad 500 007, India; e Department of Organic Chemistry, Madurai Kamaraj University, Madurai 625 021, India

## Abstract

In the title compound C_18_H_14_N_2_O, the pendant rings make dihedral angles of 66.1 (1)° and 13.9 (1) with the central ring. In the crystal, two mol­ecules form a cyclic centrosymmetric *R*_2_^2^(22) dimer through pairs of C—H⋯O bonds. These dimers are further connected into zigzag chains extending along the *b* axis through C—H⋯π and C—H⋯O inter­actions.

## Related literature

For the pharmacological and medicinal properties of the title compound, see: Baraldi *et al.* (1998 ▶); Bruno *et al.* (1990 ▶); Chen & Li (1998 ▶); Cottineau *et al.* (2002 ▶); Londershausen (1996 ▶); Mishra *et al.* (1998 ▶); Smith *et al.* (2001 ▶). For hybridization and electron delocalization, see: Beddoes *et al.* (1986 ▶); Jin *et al.* (2004 ▶). For ring and chain motifs, see: Bernstein *et al.* (1995 ▶).
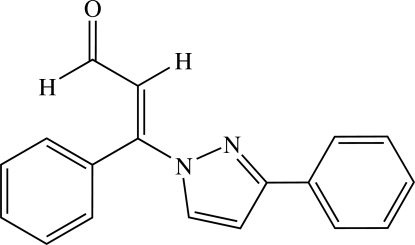


## Experimental

### 

#### Crystal data

C_18_H_14_N_2_O
*M* *_r_* = 274.31
Monoclinic, 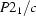

*a* = 8.6157 (7) Å
*b* = 18.0969 (12) Å
*c* = 10.0861 (6) Å
β = 113.091 (6)°
*V* = 1446.61 (19) Å^3^
*Z* = 4
Mo *K*α radiation
μ = 0.08 mm^−1^
*T* = 293 K
0.23 × 0.21 × 0.18 mm

#### Data collection

Bruker SMART APEX CCD area-detector diffractometer
Absorption correction: multi-scan (*SADABS*; Sheldrick, 2001 ▶) *T*_min_ = 0.863, *T*_max_ = 0.994
13719 measured reflections
2547 independent reflections
2262 reflections with *I* > 2σ(*I*)
*R*_int_ = 0.024

#### Refinement

*R*[*F* ^2^ > 2σ(*F* ^2^)] = 0.040
*wR*(*F* ^2^) = 0.105
*S* = 1.05
2547 reflections
190 parameters
H-atom parameters constrained
Δρ_max_ = 0.14 e Å^−3^
Δρ_min_ = −0.15 e Å^−3^

### 

Data collection: *SMART* (Bruker, 2001 ▶); cell refinement: *SAINT* (Bruker, 2001 ▶); data reduction: *SAINT*; program(s) used to solve structure: *SHELXTL/PC* (Sheldrick, 2008 ▶); program(s) used to refine structure: *SHELXTL/PC*; molecular graphics: *PLATON* (Spek, 2009 ▶); software used to prepare material for publication: *SHELXTL/PC*.

## Supplementary Material

Crystal structure: contains datablocks global, I. DOI: 10.1107/S1600536810004332/fj2268sup1.cif

Structure factors: contains datablocks I. DOI: 10.1107/S1600536810004332/fj2268Isup2.hkl

Additional supplementary materials:  crystallographic information; 3D view; checkCIF report

## Figures and Tables

**Table 1:** Hydrogen-bond geometry (Å, °) *Cg* is the centroid of the C31–C36 ring.

*D*—H⋯*A*	*D*—H	H⋯*A*	*D*⋯*A*	*D*—H⋯*A*
C35—H35⋯O1^i^	0.93	2.59	3.358 (2)	140
C13—H13⋯O1^ii^	0.93	2.66	3.360 (2)	133
C14—H14⋯*Cg*^iii^	0.93	2.95	3.703 (2)	139

Symmetry codes: (i) 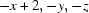; (ii) 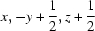; (iii) 
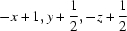
.

**Fig. 1.**
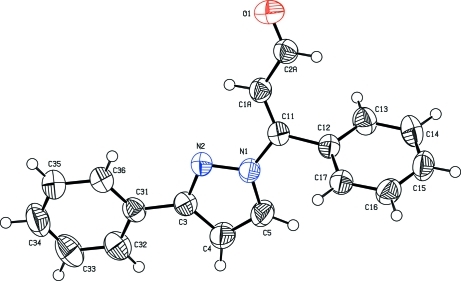
The molecular structure of the title compound with atom numbering scheme and 50% probability displacement ellipsoids.

**Fig. 2.**
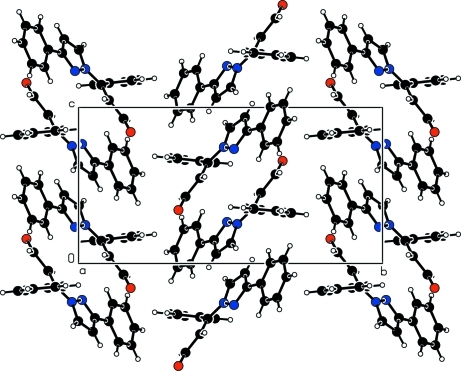
Packing diagram of the title compound viewed down the a axis.

## supplementary crystallographic information

### Comment

Pyrazole refers both to the class of simple aromatic ring organic compounds and of the heterocyclic series characterized by a 5-membered ring structure composed of three carbon atoms and two nitrogen atoms in adjacent positions and to the unsubstituted parent compound. Being so composed and having pharmacological effects on humans, they are classified as alkaloids although they are rare in nature. Pyrazole and its derivatives are successfully tested for their antifungal (Chen & Li, 1998), antihistaminic (Mishra *et al.*,1998), anti-inflammatory (Smith *et al.*, 2001), antiarrhythmic and sedative (Bruno *et al.*, 1990), hypoglycemic (Cottineau *et al.*, 2002), antiviral (Baraldi *et al.*, 1998) and pesticidal (Londershausen, 1996) activities.

An *ORTEP* plot of the molecule is shown in Fig. 1. The pyrazole ring adopts planar conformation. The phenyl rings and the plane of the pyrazole ring are making the dihedral angles of 66.1 (1)° (with C12/C17 ring) and 13.9 (1)° (with C31/C36 ring). The two phenyl rings are oriented with the dihedral angle of 79.9 (1)°. Further, pyrazole ring is making an angle of 15.8 (2)° with the propenal plane (C11,C1A,C2A,O1). The sum of the angles at N1 of the pyrazole ring (359.8 (1)°) is in accordance with *sp*^2^ hybridization (Beddoes *et al.*, 1986). The C—N bond lengths in the pyrazole ring are 1.326 (2) and 1.355 (2) Å, which are shorter than a C—N single bond length of 1.443 Å, but longer than a double bond length of 1.269 Å, (Jin *et al.*,2004), indicating electron delocalization.

The crystal packing is stabilized through moderate C—H···O, C—H···N and C—H···π interactions (Table 1). Two symmetry-related molecules form a cyclic centrosymmetric *R*_2_^2^(22) dimer through C35—H35···O1^i^ bond around the inversion centers of the unit cell (Fig 2). These dimers are connected into two zigzag chains extending along the *b* axis through C—H···π and another C—H···O interaction (Bernstein *et al.*, 1995).

### Experimental

To a mixture of 1-phenyl-1-ethanone *N*-[(*E*)-1-phenylethylidene]hydrazone (0.003 mole) and 3 ml of dimethyl formamide kept in ice bath at 0 °C, phosphorous oxycholride (0.024 mole) was added dropwise for 5 to 10 minutes. The reaction mixture was then irradiated under microwaves for 30 sec. The process of the reaction was monitored by TLC. After completion of the reaction, the reaction mixture was poured into crushed ice and extracted with dichloromethane. The organic layer was dried over anhydrous sodium sulfate. The different compound was separated by column chromatography using petroleum ether and ethyl acetate mixture as eluent. This isolated compound was recrystallized to obtain the title compound.

### Refinement

All the H atoms were positioned geometrically and refined using a riding model, with C—H = 0.93 Å and *U*_iso_(H) = 1.2 *U*_eq_ (parent atom).

### Crystal data

**Table:**

C_18_H_14_N_2_O	*F*(000) = 576
*M**_r_* = 274.31	*D*_x_ = 1.260 Mg m^−^^3^
Monoclinic, *P*2_1_/*c*	Mo *K*α radiation, λ = 0.71073 Å
Hall symbol: -P 2ybc	Cell parameters from 5126 reflections
*a* = 8.6157 (7) Å	θ = 1.7–27.6°
*b* = 18.0969 (12) Å	µ = 0.08 mm^−^^1^
*c* = 10.0861 (6) Å	*T* = 293 K
β = 113.091 (6)°	Block, colourless
*V* = 1446.61 (19) Å^3^	0.23 × 0.21 × 0.18 mm
*Z* = 4	

### Data collection

**Table:**

Bruker SMART APEX CCD area-detector diffractometer	2547 independent reflections
Radiation source: fine-focus sealed tube	2262 reflections with *I* > 2σ(*I*)
graphite	*R*_int_ = 0.024
ω scans	θ_max_ = 25.0°, θ_min_ = 2.3°
Absorption correction: multi-scan (*SADABS*; Sheldrick, 2001)	*h* = −10→10
*T*_min_ = 0.863, *T*_max_ = 0.994	*k* = −21→21
13719 measured reflections	*l* = −11→11

### Refinement

**Table:**

Refinement on *F*^2^	Primary atom site location: structure-invariant direct methods
Least-squares matrix: full	Secondary atom site location: difference Fourier map
*R*[*F*^2^ > 2σ(*F*^2^)] = 0.040	Hydrogen site location: inferred from neighbouring sites
*wR*(*F*^2^) = 0.105	H-atom parameters constrained
*S* = 1.05	*w* = 1/[σ^2^(*F*_o_^2^) + (0.0509*P*)^2^ + 0.2744*P*] where *P* = (*F*_o_^2^ + 2*F*_c_^2^)/3
2547 reflections	(Δ/σ)_max_ = 0.001
190 parameters	Δρ_max_ = 0.14 e Å^−^^3^
0 restraints	Δρ_min_ = −0.14 e Å^−^^3^

### Special details

**Table:**

Geometry. All e.s.d.'s (except the e.s.d. in the dihedral angle between two l.s. planes) are estimated using the full covariance matrix. The cell e.s.d.'s are taken into account individually in the estimation of e.s.d.'s in distances, angles and torsion angles; correlations between e.s.d.'s in cell parameters are only used when they are defined by crystal symmetry. An approximate (isotropic) treatment of cell e.s.d.'s is used for estimating e.s.d.'s involving l.s. planes.
Refinement. Refinement of *F*^2^ against ALL reflections. The weighted *R*-factor *wR* and goodness of fit *S* are based on *F*^2^, conventional *R*-factors *R* are based on *F*, with *F* set to zero for negative *F*^2^. The threshold expression of *F*^2^ > σ(*F*^2^) is used only for calculating *R*-factors(gt) *etc*. and is not relevant to the choice of reflections for refinement. *R*-factors based on *F*^2^ are statistically about twice as large as those based on *F*, and *R*- factors based on ALL data will be even larger.

### Fractional atomic coordinates and isotropic or equivalent isotropic displacement parameters (Å^2^)

**Table:**

	*x*	*y*	*z*	*U*_iso_*/*U*_eq_	
N1	0.61426 (14)	0.02209 (6)	0.24676 (12)	0.0463 (3)	
N2	0.74924 (13)	−0.01152 (6)	0.23393 (12)	0.0447 (3)	
C3	0.81474 (17)	−0.05400 (8)	0.34980 (14)	0.0467 (3)	
C4	0.7236 (2)	−0.04724 (11)	0.43775 (17)	0.0663 (5)	
H4	0.7448	−0.0712	0.5247	0.080*	
C5	0.5990 (2)	0.00119 (10)	0.37010 (17)	0.0649 (5)	
H5	0.5172	0.0174	0.4022	0.078*	
C11	0.52083 (16)	0.07517 (7)	0.14569 (14)	0.0432 (3)	
C12	0.36253 (17)	0.10037 (7)	0.15681 (14)	0.0442 (3)	
C13	0.3380 (2)	0.17479 (9)	0.17313 (18)	0.0573 (4)	
H13	0.4230	0.2085	0.1821	0.069*	
C14	0.1874 (2)	0.19915 (10)	0.17617 (19)	0.0690 (5)	
H14	0.1716	0.2493	0.1871	0.083*	
C15	0.0613 (2)	0.14992 (10)	0.16317 (18)	0.0653 (5)	
H15	−0.0406	0.1667	0.1633	0.078*	
C16	0.08564 (19)	0.07612 (10)	0.14998 (17)	0.0595 (4)	
H16	0.0008	0.0427	0.1428	0.071*	
C17	0.23541 (18)	0.05101 (8)	0.14726 (15)	0.0510 (4)	
H17	0.2513	0.0007	0.1390	0.061*	
C31	0.96351 (17)	−0.09984 (7)	0.37111 (16)	0.0478 (4)	
C32	1.0553 (2)	−0.13183 (9)	0.50393 (17)	0.0626 (4)	
H32	1.0215	−0.1250	0.5801	0.075*	
C33	1.1966 (2)	−0.17376 (10)	0.5237 (2)	0.0757 (5)	
H33	1.2567	−0.1955	0.6128	0.091*	
C34	1.2482 (2)	−0.18350 (11)	0.4134 (3)	0.0815 (6)	
H34	1.3454	−0.2106	0.4281	0.098*	
C35	1.1569 (2)	−0.15340 (10)	0.2805 (2)	0.0772 (5)	
H35	1.1913	−0.1608	0.2048	0.093*	
C36	1.0147 (2)	−0.11233 (9)	0.25925 (19)	0.0598 (4)	
H36	0.9524	−0.0928	0.1686	0.072*	
C1A	0.57622 (17)	0.10007 (8)	0.04689 (15)	0.0483 (3)	
H1A	0.6831	0.0854	0.0552	0.058*	
C2A	0.48155 (19)	0.14796 (8)	−0.07132 (16)	0.0524 (4)	
H2A	0.3733	0.1617	−0.0819	0.063*	
O1	0.53501 (16)	0.17125 (7)	−0.15736 (13)	0.0747 (4)	

### Atomic displacement parameters (Å^2^)

**Table:**

	*U*^11^	*U*^22^	*U*^33^	*U*^12^	*U*^13^	*U*^23^
N1	0.0437 (6)	0.0521 (7)	0.0449 (6)	0.0073 (5)	0.0193 (5)	0.0021 (5)
N2	0.0409 (6)	0.0464 (7)	0.0471 (7)	0.0035 (5)	0.0174 (5)	−0.0006 (5)
C3	0.0451 (8)	0.0474 (8)	0.0438 (8)	0.0006 (6)	0.0133 (6)	0.0000 (6)
C4	0.0683 (10)	0.0862 (12)	0.0498 (9)	0.0222 (9)	0.0291 (8)	0.0186 (8)
C5	0.0647 (10)	0.0859 (12)	0.0543 (9)	0.0226 (9)	0.0344 (8)	0.0143 (8)
C11	0.0404 (7)	0.0424 (7)	0.0439 (7)	−0.0005 (6)	0.0136 (6)	−0.0044 (6)
C12	0.0424 (7)	0.0479 (8)	0.0406 (7)	0.0033 (6)	0.0147 (6)	−0.0014 (6)
C13	0.0544 (9)	0.0491 (8)	0.0668 (10)	0.0023 (7)	0.0221 (8)	−0.0055 (7)
C14	0.0715 (11)	0.0548 (10)	0.0816 (12)	0.0193 (8)	0.0312 (9)	−0.0035 (8)
C15	0.0508 (9)	0.0807 (12)	0.0679 (11)	0.0188 (8)	0.0270 (8)	0.0051 (9)
C16	0.0470 (8)	0.0715 (11)	0.0627 (10)	0.0010 (7)	0.0244 (7)	0.0055 (8)
C17	0.0495 (8)	0.0499 (8)	0.0554 (9)	0.0015 (6)	0.0227 (7)	−0.0002 (7)
C31	0.0434 (8)	0.0411 (7)	0.0539 (8)	−0.0011 (6)	0.0137 (6)	−0.0006 (6)
C32	0.0595 (10)	0.0632 (10)	0.0544 (9)	0.0050 (8)	0.0106 (8)	0.0007 (7)
C33	0.0610 (11)	0.0675 (11)	0.0761 (12)	0.0148 (9)	0.0025 (9)	0.0085 (9)
C34	0.0601 (11)	0.0664 (11)	0.1134 (17)	0.0207 (9)	0.0290 (11)	0.0097 (11)
C35	0.0775 (12)	0.0679 (11)	0.1003 (15)	0.0211 (9)	0.0500 (11)	0.0127 (10)
C36	0.0612 (10)	0.0552 (9)	0.0675 (10)	0.0123 (7)	0.0301 (8)	0.0106 (7)
C1A	0.0406 (7)	0.0501 (8)	0.0541 (8)	0.0016 (6)	0.0186 (6)	0.0003 (6)
C2A	0.0518 (8)	0.0488 (8)	0.0570 (9)	−0.0003 (7)	0.0216 (7)	0.0008 (7)
O1	0.0825 (9)	0.0766 (8)	0.0753 (8)	0.0041 (6)	0.0420 (7)	0.0219 (6)

### Geometric parameters (Å, °)

**Table:**

N1—C5	1.3552 (19)	C16—C17	1.378 (2)
N1—N2	1.3627 (15)	C16—H16	0.9300
N1—C11	1.4023 (18)	C17—H17	0.9300
N2—C3	1.3259 (17)	C31—C36	1.382 (2)
C3—C4	1.402 (2)	C31—C32	1.387 (2)
C3—C31	1.470 (2)	C32—C33	1.380 (2)
C4—C5	1.346 (2)	C32—H32	0.9300
C4—H4	0.9300	C33—C34	1.363 (3)
C5—H5	0.9300	C33—H33	0.9300
C11—C1A	1.340 (2)	C34—C35	1.373 (3)
C11—C12	1.4835 (18)	C34—H34	0.9300
C12—C13	1.383 (2)	C35—C36	1.376 (2)
C12—C17	1.387 (2)	C35—H35	0.9300
C13—C14	1.381 (2)	C36—H36	0.9300
C13—H13	0.9300	C1A—C2A	1.440 (2)
C14—C15	1.371 (3)	C1A—H1A	0.9300
C14—H14	0.9300	C2A—O1	1.2068 (18)
C15—C16	1.366 (2)	C2A—H2A	0.9300
C15—H15	0.9300		
			
C5—N1—N2	110.89 (11)	C15—C16—H16	119.8
C5—N1—C11	128.59 (12)	C17—C16—H16	119.8
N2—N1—C11	120.31 (11)	C16—C17—C12	120.40 (14)
C3—N2—N1	105.02 (11)	C16—C17—H17	119.8
N2—C3—C4	110.90 (13)	C12—C17—H17	119.8
N2—C3—C31	120.10 (13)	C36—C31—C32	118.46 (14)
C4—C3—C31	129.01 (13)	C36—C31—C3	120.81 (13)
C5—C4—C3	105.64 (14)	C32—C31—C3	120.73 (14)
C5—C4—H4	127.2	C33—C32—C31	120.37 (17)
C3—C4—H4	127.2	C33—C32—H32	119.8
C4—C5—N1	107.55 (14)	C31—C32—H32	119.8
C4—C5—H5	126.2	C34—C33—C32	120.31 (17)
N1—C5—H5	126.2	C34—C33—H33	119.8
C1A—C11—N1	120.03 (12)	C32—C33—H33	119.8
C1A—C11—C12	123.97 (13)	C33—C34—C35	120.04 (17)
N1—C11—C12	116.00 (12)	C33—C34—H34	120.0
C13—C12—C17	118.80 (13)	C35—C34—H34	120.0
C13—C12—C11	119.78 (13)	C34—C35—C36	120.04 (18)
C17—C12—C11	121.40 (12)	C34—C35—H35	120.0
C14—C13—C12	120.15 (15)	C36—C35—H35	120.0
C14—C13—H13	119.9	C35—C36—C31	120.73 (16)
C12—C13—H13	119.9	C35—C36—H36	119.6
C15—C14—C13	120.42 (16)	C31—C36—H36	119.6
C15—C14—H14	119.8	C11—C1A—C2A	124.51 (13)
C13—C14—H14	119.8	C11—C1A—H1A	117.7
C16—C15—C14	119.87 (15)	C2A—C1A—H1A	117.7
C16—C15—H15	120.1	O1—C2A—C1A	123.61 (14)
C14—C15—H15	120.1	O1—C2A—H2A	118.2
C15—C16—C17	120.33 (16)	C1A—C2A—H2A	118.2
			
C5—N1—N2—C3	0.88 (16)	C13—C14—C15—C16	1.3 (3)
C11—N1—N2—C3	175.96 (12)	C14—C15—C16—C17	−1.1 (3)
N1—N2—C3—C4	−0.64 (16)	C15—C16—C17—C12	−0.5 (2)
N1—N2—C3—C31	179.26 (12)	C13—C12—C17—C16	1.8 (2)
N2—C3—C4—C5	0.2 (2)	C11—C12—C17—C16	−176.44 (13)
C31—C3—C4—C5	−179.71 (15)	N2—C3—C31—C36	−14.4 (2)
C3—C4—C5—N1	0.4 (2)	C4—C3—C31—C36	165.51 (16)
N2—N1—C5—C4	−0.8 (2)	N2—C3—C31—C32	165.91 (14)
C11—N1—C5—C4	−175.36 (15)	C4—C3—C31—C32	−14.2 (2)
C5—N1—C11—C1A	165.51 (15)	C36—C31—C32—C33	1.4 (2)
N2—N1—C11—C1A	−8.60 (19)	C3—C31—C32—C33	−178.88 (15)
C5—N1—C11—C12	−14.5 (2)	C31—C32—C33—C34	0.7 (3)
N2—N1—C11—C12	171.43 (11)	C32—C33—C34—C35	−1.9 (3)
C1A—C11—C12—C13	−56.2 (2)	C33—C34—C35—C36	1.1 (3)
N1—C11—C12—C13	123.79 (15)	C34—C35—C36—C31	1.1 (3)
C1A—C11—C12—C17	122.03 (16)	C32—C31—C36—C35	−2.3 (2)
N1—C11—C12—C17	−58.00 (17)	C3—C31—C36—C35	178.02 (15)
C17—C12—C13—C14	−1.6 (2)	N1—C11—C1A—C2A	172.66 (13)
C11—C12—C13—C14	176.68 (14)	C12—C11—C1A—C2A	−7.4 (2)
C12—C13—C14—C15	0.0 (3)	C11—C1A—C2A—O1	178.19 (15)

### Hydrogen-bond geometry (Å, °)

**Table:**

Cg is the centroid of the C31–C36 ring.

**Table:**

*D*—H···*A*	*D*—H	H···*A*	*D*···*A*	*D*—H···*A*
C35—H35···O1^i^	0.93	2.59	3.358 (2)	140
C13—H13···O1^ii^	0.93	2.66	3.360 (2)	133
C14—H14···Cg^iii^	0.93	2.95	3.703 (2)	139

Symmetry codes: (i) −*x*+2, −*y*, −*z*; (ii) *x*, −*y*+1/2, *z*+1/2; (iii) −*x*+1, *y*+1/2, −*z*+1/2.
